# The Enigma of Retroperitoneal Fibrosis: Clinical Implications and Diagnostic and Therapeutic Challenges

**DOI:** 10.7759/cureus.74499

**Published:** 2024-11-26

**Authors:** Gowri Renganathan, Sudhagar Thangarasu, Nibesh Pathak, Vamsi K Kunam, Zeeshan Afzal

**Affiliations:** 1 Department of Internal Medicine, Texas Tech University Health Sciences Center Paul L. Foster School of Medicine, El Paso, USA; 2 Internal Medicine, Tribhuvan University, Kathmandu, NPL; 3 Interventional Radiology, The Hospitals of Providence Transmountain, El Paso, USA; 4 Department of Internal Medicine/Rheumatology, Texas Tech University Health Sciences Center Paul L. Foster School of Medicine, El Paso, USA

**Keywords:** corticosteroids, deep venous thrombosis (dvt), inferior vena cava syndrome, normal serum igg4 levels, retroperitoneal fibrosis

## Abstract

Retroperitoneal fibrosis (RPF) is a rare disease with a nonspecific presentation. RPF can be classified into Idiopathic, the most common, or secondary due to malignancy and various medications resulting in chronic inflammation and fibrosis in the retroperitoneum. The complications arise due to the compression of structures in the retroperitoneum. The most common presentations are constitutional symptoms, abdominal pain, and renal insufficiency due to ureteral obstruction. Venous thrombosis or claudication on presentation is rare. Diagnosis and treatment remain challenging due to the lack of standard diagnostic or treatment protocols. Our patient presented with symptoms of acute deep vein thrombosis (DVT) with varices on the abdomen and mild bilateral hydronephrosis. A CT scan revealed a retroperitoneal mass, which was confirmed to be RPF by biopsy. Relevant laboratory tests, including IgG4, were negative. High-dose corticosteroid therapy reduced the inflammatory markers and the size of the retroperitoneal mass.

## Introduction

Retroperitoneal fibrosis (RPF) is a chronic inflammatory condition affecting the retroperitoneum, marked by the development of fibro-sclerotic tissue within retroperitoneal structures. Idiopathic retroperitoneal fibrosis (IRF) constitutes around two-thirds of RPF cases, with the remaining third attributed to secondary causes such as medications, neoplasms, radiotherapy, infections, surgeries, and malignancies. RPF typically manifests with a gradual clinical onset, presenting symptoms such as flank, back, or abdominal pain, along with constitutional symptoms like malaise, fever, anorexia, and weight loss. Laboratory findings often reveal elevated erythrocyte sedimentation rate, increased C-reactive protein levels, elevated urea and creatinine levels, anemia, polyclonal hypergammaglobulinemia, elevated alkaline phosphatase, and the presence of antinuclear antibodies. Venous compression, particularly of the inferior vena cava, is common in RPF and may result in lower limb edema, potentially exacerbated by lymphatic compression. The gradual encasement of veins often leads to collateral circulation, mitigating complications such as inferior vena cava syndrome, deep vein thrombosis, and pulmonary embolism. The diagnosis is based on computed tomography or magnetic resonance imaging and confirmed by biopsy.

## Case presentation

A 39-year-old male presented to our Emergency Department with the chief complaint of bilateral lower extremity swelling for three days associated with dyspnea on exertion. He denied pain, numbness, tingling, chest pain, or palpitation. He had no urinary symptoms or hematuria. There is no history of recent surgery, prolonged immobilization, and malignancy. His past medical history included hyperlipidemia and hypertension, which was well controlled on losartan. He works from home and his social habits were smoking 20 pack-year, about one drink of alcohol per day, and snorting cocaine once a month; his last use was three months before presentation. His family history is significant for breast cancer in his mother and lung cancer and cirrhosis in his father with no familial history of any autoimmune or hematological conditions. His primary care physician diagnosed him with hydrocele and varicose veins in the chest and abdomen.

**Figure 1:**
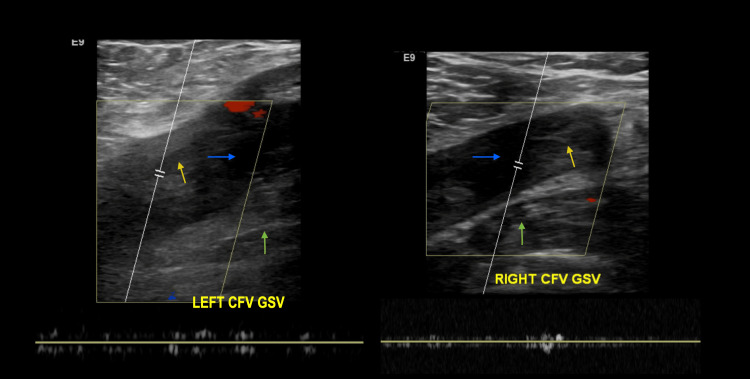
Ultrasound of the bilateral lower extremities showing occlusive echogenic filling defects (yellow arrow) in bilateral common femoral veins with no flow, consistent with DVT. CFV: common femoral vein (blue arrow), GSV: greater saphenous vein (green arrow), DVT: deep vein thrombosis

On examination, the patient was comfortable with no apparent distress. His vitals were stable. He had bilateral pedal edema (2+) extending up to the knees, and his right lower limb was tender to palpation. Dilated veins were noticed on the anterior chest and upper abdomen. On genitourinary examination, he had a right-sided hydrocele. His chest was clear to auscultation. With an early suspicion of pulmonary embolism and DVT, a duplex ultrasound exam of the lower limb was done, which showed occlusive echogenic filling defects in bilateral common femoral veins, with no flow consistent with DVT (Figure 1).

**Figure 2:**
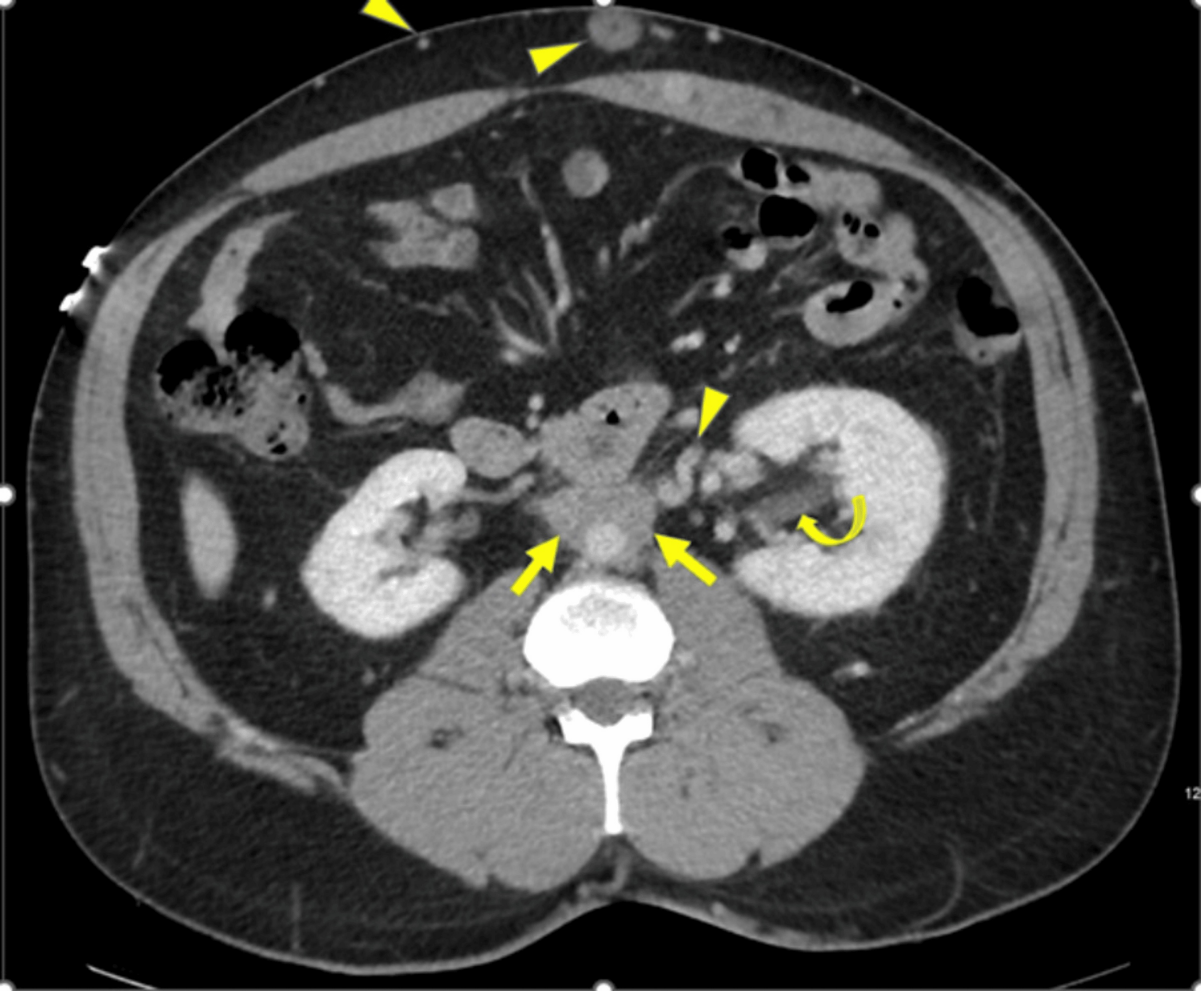
Axial CT image shows periaortic soft tissue (bold arrows), severe compression of IVC (not seen) with multiple retroperitoneal and subcutaneous venous collaterals (arrowheads), compressing the ureters and left hydronephrosis (curved arrow) CT-Computed Tomography IVC- Inferior Vena Cava

**Figure 3:**
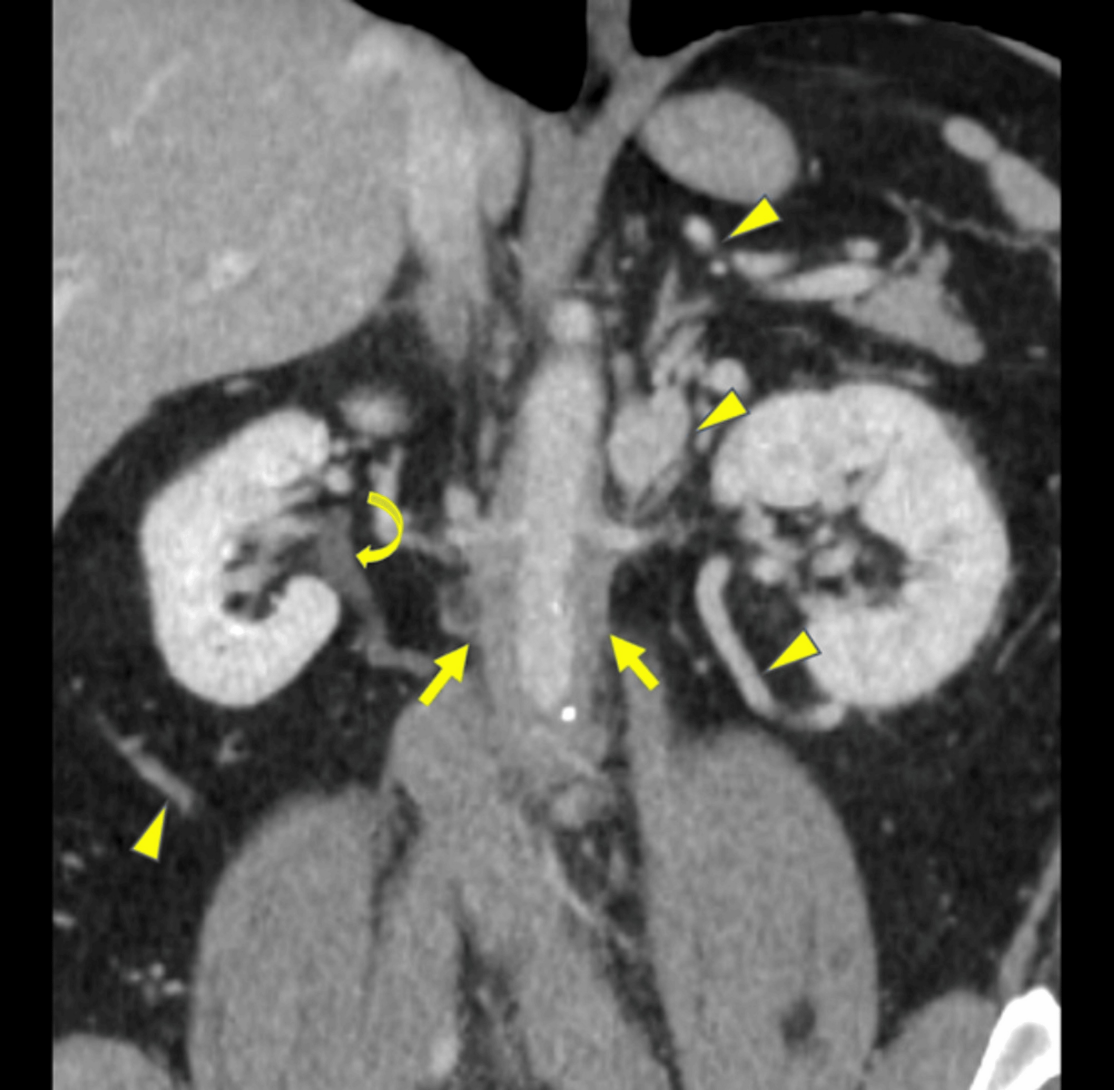
Coronal CT image shows periaortic soft tissue (bold arrows), severe compression of IVC (not seen) with multiple retroperitoneal and subcutaneous venous collaterals (arrowheads), compressing the ureters and right hydronephrosis (curved arrow)

There was no evidence of pulmonary embolism on the computed tomography (CT) angiogram of the chest. Still, there was prominence of azygous vein and varicosity in the anterior chest wall and abdomen. CT abdomen showed soft tissue density surrounding the mid and distal abdominal aorta. External and common iliac vessels were dilated. Infra-renal vena cava was not visualized and was not patent (Figures 2, 3). Extensive varices were noted in the anterior abdominal wall and left anterior rectus muscle. Therapeutic anticoagulation with heparin was started and transitioned later to oral apixaban on the day of discharge.

Initial blood investigations (Table 1) such as hemogram, liver and renal function tests, and urine analysis were within normal limits. The urine drug screen is negative. The inflammatory marker levels (erythrocyte sedimentation rate and C-reactive protein) were elevated. Autoimmune markers like ANA, ANCA IgG4, antiphospholipid antibody, b2 glycoprotein, cardiolipin, and rheumatoid factor were negative. Screening tests for tuberculosis, hepatitis B and C, and human immunodeficiency virus (HIV) were negative. Rapid plasma reagin (RPR) for syphilis was negative.

**Table 1:** Laboratory findings

Investigation	Patient value	Reference value (normal)
Hemoglobin	14 mg/dl	Female: 12-16g/dl; male:14-18g/dl
Platelets	189,000	150000-450000 /microL
Blood urea nitrogen (BUN)	15	8-20 mg/dl
Creatinine	1.1	Female: 0.5-1.1 mg/dl; male:0.7-1.3 mg/dl
C-reactive protein (CRP)	54 mg/dl	≤0.8mg/dl
Erythrocyte sedimentation rate	79 mm/hr	Female: 0-20 mm/hour; male: 0-15 mm/hour

**Figure 4:**
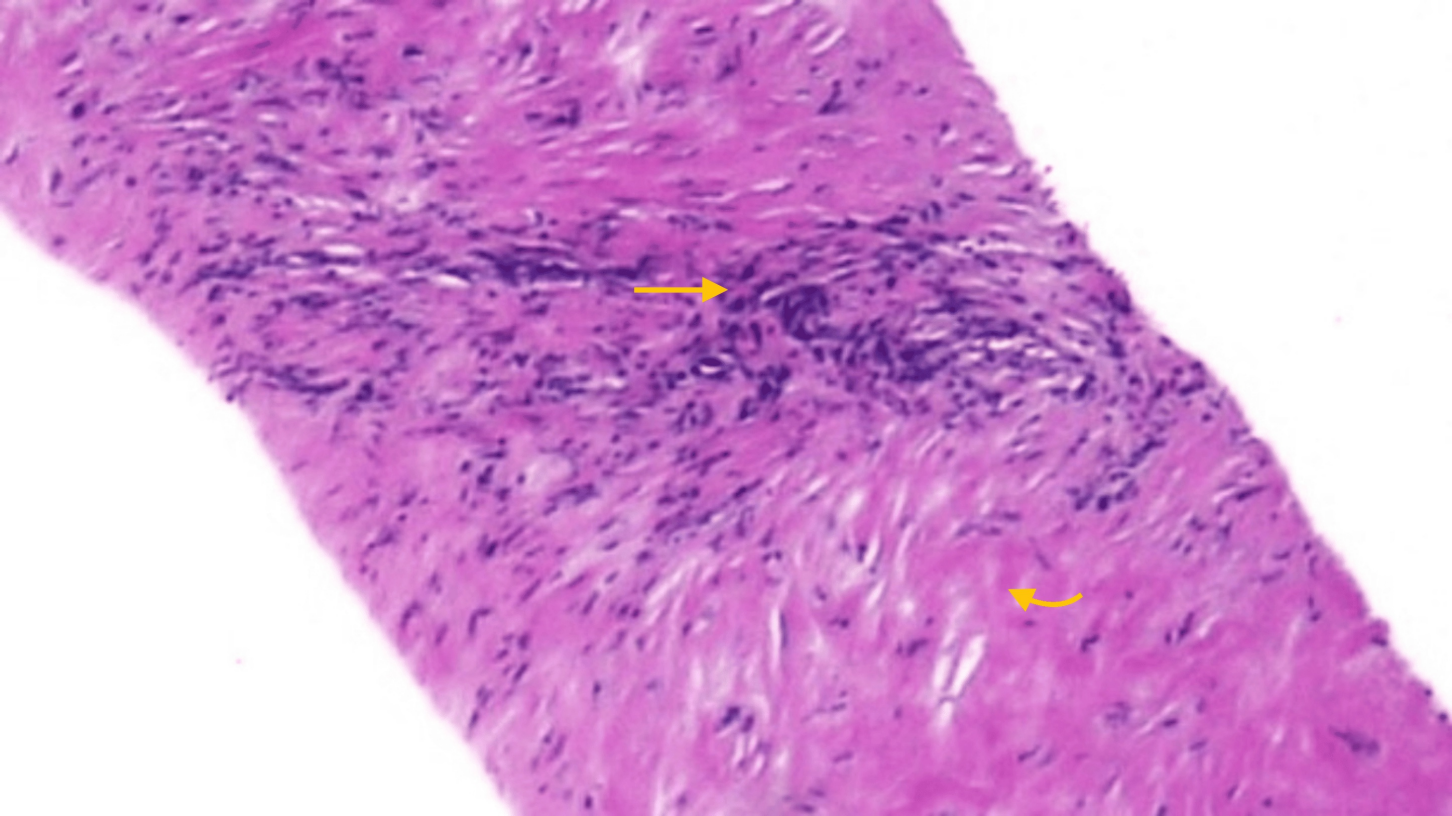
Histopathological examination showing dense fibrosis (curved arrow) with foci of chronic inflammation (arrow).

Subsequently, an interventional radiologist performed a biopsy of the retroperitoneal mass. Histopathological examination (Figure 4) showed dense fibrosis with foci of chronic inflammation, features suggestive of RPF. Since no attributable cause was identified, a diagnosis of IRPF was made.

The patient continued taking apixaban and was prescribed high-dose systemic steroids of 1 mg/kg, which were tapered slowly over several months to low-dose maintenance of 5-7.5 mg daily. Inflammatory markers have normalized, as shown in Table 2, within a month of initiating treatment with steroids. Follow-up was done using PET/CT scans, which showed improvement in paraaortic soft tissue density just above the aortoiliac bifurcation and along the anterior margin of the aorta and, specifically no abnormal paraaortic FDG hypermetabolic lesion.

**Table 2:** Steroid tapering schedule ESR: erythrocyte sedimentation rate, CRP: C-reactive protein

Date	ESR (mm/hr)	CRP (mg/dl)	Steroid dose (prednisone)
10/22	79	54.6	60 mg daily x 4 weeks
11/22	2	0.9	Tapered by 5 mg every 2 week
02/23	6	0.9	20 mg daily
04/23	6	1	15 mg daily
05/23	6	1.8	12.5 mg x 4 weeks, then 10 mg daily
08/23	2	1.5	7.5 mg x 4 weeks, then 5 mg daily

## Discussion

RPF is a chronic inflammatory condition affecting the retroperitoneum, marked by the development of fibro-sclerotic tissue within retroperitoneal structures [1]. IRF constitutes around two-thirds of RPF cases, with the remaining third attributed to secondary causes such as medications, neoplasms, radiotherapy, infections, surgeries, and malignancies [2,3]. IRF is rare, with a prevalence of 1.4 cases per 100,000 people and an estimated annual incidence of 0.1-1.3 cases per 100,000 people, typically affecting individuals aged 55 to 60, with a male-to-female ratio of 2:1-3:1 and long-term mortality rates ranging from 3.3% to 7.3% [4,5]. Differential diagnoses for RPF include retroperitoneal lymphoma, sarcoma, secondary metastasis, tuberculosis, pelvic actinomyces, and retroperitoneal Erdheim-Chester disease. It has been associated with the use of some drugs, such as anti-tumor necrosis factor, ergot alkaloids, and dopamine agonists. RPF is sometimes called “chronic peri-aortitis” because it involves the adventitia of the abdominal and thoracic aorta, iliac arteries, and surrounding retroperitoneum [6].

The disease manifests with systemic symptoms like fatigue, anorexia, and weight loss, usually accompanied by back, flank, or abdomen pain. It commonly presents with urological symptoms, including scrotal discomfort, often accompanied by hydrocele (as in our case), varicocele, retrograde ejaculation, and erectile dysfunction [3]. The disease usually causes medial ureteral deviation and frequent ureteral obstruction; our patient had mild bilateral hydronephrosis, but no urinary symptoms or hematuria, and his kidney function was normal. IRF can extend to the renal vascular peduncle, leading to compression of renal veins, which typically progresses slowly and facilitates the development of collateral circulation, as well as compression of renal arteries, resulting in reno-vascular hypertension, with patients presenting as new onset hypertension in up to one-third of cases [7]. Venous compression, particularly of the inferior vena cava, is frequent and can lead to lower limb edema similar to our patient’s presentation, possibly augmented by lymphatic compression as well. Due to the gradual progression of venous encasement, collateral circulation often develops, resulting in rare occurrences of inferior vena cava syndrome, deep vein thrombosis, and pulmonary embolism [4,7,8]. However, this patient had acute DVT at presentation. According to a retrospective study in Europe, out of 213 patients with IRF, 18 had IVC occlusion, out of which one had acute DVT [9].

In most patients at presentation, acute phase reactants (APRs) such as ESR and CRP levels are elevated. They are commonly used to monitor disease activity, as we did for our patient. While elevated baseline APR are associated with more symptomatic disease, they poorly predict therapy response, do not correlate with mass regression, and relapses frequently occur even when APR is normal [8]. The serum concentration of CCL18 signifies fibroinflammatory activity and disease extent in patients with chronic peri-aortitis [3].

IgG4-related disease comprises a significant portion (57%) of patients with IRF [10]. In about two-thirds of patients with IgG-related disease, serum IgG4 levels exceed the upper normal limit. By contrast, approximately 30% of patients diagnosed with IgG4-related disease have normal serum IgG4 levels, indicating that a normal serum IgG4 level does not rule out the disease [11]. Patients with IRF show a higher prevalence of anti-thyroperoxidase antibodies than healthy individuals. During follow-up, a quarter of IRF patients developed hypothyroidism requiring L-thyroxine treatment, emphasizing the importance of monitoring thyroid function in IRF patients [12]. Peripheral inflammatory arthritis, such as rheumatoid arthritis (RA) or RA-like forms, may occur alongside chronic peri-aortitis (CP), with documented cases showing associations with spondyloarthritis like ankylosing spondylitis (AS), ANCA-associated vasculitis, psoriasis, and membranous glomerulonephritis [3]. However, thyroid function was normal, and the autoimmune panel was negative for our patient. The disease's pathogenesis is multifactorial. Remarkably, smoking and asbestos exhibited a multiplicative effect on disease risk, resulting in a significantly higher odds ratio in co-exposed subjects [8].

CT or magnetic resonance imaging (MRI) is the main diagnostic modality of RPF. RPF typically manifests as a paraspinal mass in the retroperitoneum, appearing isodense to surrounding muscle and encasing structures like the aorta, IVC, and ureters, often leading to hydro-uretero-nephrosis and the degree of soft tissue enhancement on CT scans correlates with disease activity. In MRI, RPF shows low intensity in T1-weighted images and variable, often high intensity in active stages in T2-weighted images, although discerning between benign and malignant causes based on factors like aortic elevation, soft-tissue distribution, marginal lobulations, and contrast enhancement remains challenging [13].

118F-fludeoxyglucose-18 (FDG) positron emission tomography (PET) scan is valuable for assessing RPF activity, showing increased radiotracer uptake in metabolically active RPF, irrespective of its benign or malignant nature. It can also help detect conditions like multifocal fibrosclerosis, occult neoplasms, or RPF-related infectious conditions. This technique may also be valuable in follow-up, as increasing radiotracer uptake indicates inflammatory relapse.

Glucocorticoids are considered the primary treatment for RPF, although the ideal dosage and duration remain uncertain. However, some articles do not recommend its use in patients without ureteral obstruction [14]. Some vascular or urinary tract obstructions might require surgical interventions. They lead to swift alleviation of symptoms and decreased APR levels, frequently resulting in disease shrinkage. Remission is normally defined by a decline in APR level, decreased symptoms, resolving hydronephrosis, and a reduction in radiographic abnormalities. Remission rates following steroid therapy range between 75% and 95%, with an average reduction in mass thickness of approximately 50% [5,15]. Tamoxifen, an anti-estrogen agent, has been proposed as an alternative to glucocorticoids for RPF treatment, especially in patients with steroid-related toxicity or contraindications [5]. Immunosuppressants such as mycophenolate mofetil are preferred for their tolerability, especially in patients with renal insufficiency. While effective initially, cyclophosphamide is not the first-line treatment, and methotrexate in combination with corticosteroids is utilized for relapsing cases. In refractory situations, biological agents like rituximab and tocilizumab have demonstrated efficacy [3,14].

IRF is indeed a chronic-relapsing disorder, with relapse rates of up to 72%. The patients who achieve remission must be carefully followed using laboratory tests, periodic ultrasound scans (to monitor hydronephrosis and aneurysmal dilatation of blood vessels), and CT/MRI studies (to define the size and morphologic changes of RPF accurately) to allow early detection of relapses [15].

## Conclusions

RPF manifests with diverse presentations, requiring clinicians to maintain a high index of suspicion. Acute DVT as a presentation, as seen in our case, is rare due to collateral formation. Diagnosis usually involves CT/MRI and biopsy confirmation, with PET scans assisting in follow-up. Secondary causes need to be ruled out. About 30% of IRF cases, like ours, show normal IgG4 levels. Corticosteroids continue to be the primary treatment, significantly reducing the size of the mass and the inflammatory markers.
